# Association Between Mental Health Conditions and Care Fragmentation: A National Study of High-Risk Older Veterans

**DOI:** 10.1093/geroni/igaa057.1202

**Published:** 2020-12-16

**Authors:** Ranak Trivedi, Fernanda Rossi, Sarah Javier, Liberty Greene, Sara Singer, Megan Vanneman, Mary Goldstein, Donna Zulman

**Affiliations:** 1 VA Palo Alto/Stanford University, Menlo Park, California, United States; 2 VA Palo Alto Health Care System, Menlo Park, California, United States; 3 VA Palo Alto Healthcare System, Menlo Park, California, United States; 4 Stanford University, Stanford, California, United States; 5 University of Utah School of Medicine, Salt Lake City, Utah, United States; 6 Stanford University, Palo Alto, California, United States

## Abstract

Fragmented healthcare causes information loss, duplicative tests, and unwieldy self-care regimens. These challenges may be amplified among older, high-risk patients with co-occurring mental health conditions (MHC). We compared healthcare fragmentation for chronic physical conditions among Veterans with and without MHC (depression, PTSD, schizophrenia, bipolar disorder, anxiety, personality disorder, or psychosis based on ICD-9 codes). Sample included Veterans who were □65y, at high risk for 1-year hospitalization, and had □4 non-MHC visits during FY14. Visits were covered by Veterans Affairs (VA), VA-purchased care (both from VA Corporate Data Warehouse), or Medicare Parts A/B (claims data from VA Information Resource Center). Outcomes were two fragmentation measures calculated in FY15: 1) non-mental health provider count, where a higher number indicates more fragmentation, and 2) Usual Provider of Care (UPC), the proportion of care with the most frequently seen provider, where a higher number indicates less fragmentation. We used Poisson regression and GLM with binomial distribution and logit link to test the association between MHC status and fragmentation, controlling for sociodemographic characteristics (e.g., age), medical comorbidity, and driving distance to VA. Of the 125,481 Veterans included, 47.3% had 1+ MHC. Compared to older, high-risk Veterans without MHC, those with MHC saw fewer providers (pseudo R2 = 0.02) and had a higher UPC (more concentrated care; OR = 1.07). Within the VA, older, high-risk Veterans with MHC do not experience greater healthcare fragmentation. Further research is needed to determine if this is due to different needs, underuse, or appropriate use of healthcare across the groups.

